# Applied Machine Learning for IIoT and Smart Production—Methods to Improve Production Quality, Safety and Sustainability

**DOI:** 10.3390/s22239148

**Published:** 2022-11-25

**Authors:** Attila Frankó, Gergely Hollósi, Dániel Ficzere, Pal Varga

**Affiliations:** Department of Telecommunications and Media Informatics, Faculty of Electrical Engineering and Informatics, Budapest University of Technology and Economics, Muegyetem rkp. 3., H-1111 Budapest, Hungary

**Keywords:** machine learning, industry 4.0, industrial IoT, safety, security, asset localization, quality control, proactive maintenance, fault detection, prognostics

## Abstract

Industrial IoT (IIoT) has revolutionized production by making data available to stakeholders at many levels much faster, with much greater granularity than ever before. When it comes to smart production, the aim of analyzing the collected data is usually to achieve greater efficiency in general, which includes increasing production but decreasing waste and using less energy. Furthermore, the boost in communication provided by IIoT requires special attention to increased levels of safety and security. The growth in machine learning (ML) capabilities in the last few years has affected smart production in many ways. The current paper provides an overview of applying various machine learning techniques for IIoT, smart production, and maintenance, especially in terms of safety, security, asset localization, quality assurance and sustainability aspects. The approach of the paper is to provide a comprehensive overview on the ML methods from an application point of view, hence each domain—namely security and safety, asset localization, quality control, maintenance—has a dedicated chapter, with a concluding table on the typical ML techniques and the related references. The paper summarizes lessons learned, and identifies research gaps and directions for future work.

## 1. Introduction

The motivations for industrial data processing have been the same for decades, namely to increase the revenue of investment by improving efficiency (i.e., by increasing productivity, and decreasing scrap, waste and energy usage), extending system lifetime, as well as enhancing safety and security. Sustainability has become yet another focal point of modern industry.

**Figure 1:**
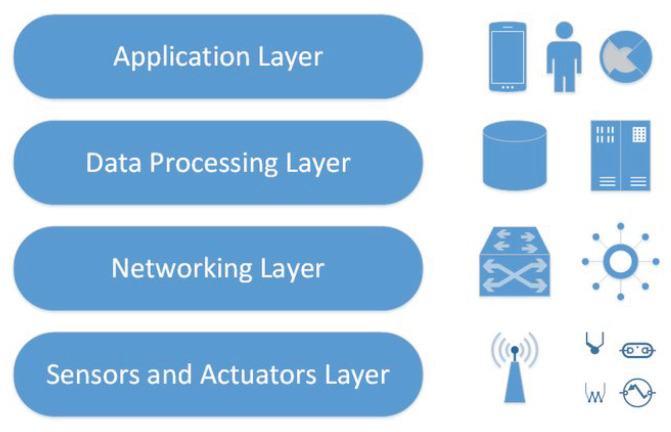
The architectural layers of IIoT systems [1].

As people acknowledged the necessity of distributed data collection and massive data processing in various industrial areas, the research and innovation domain of IIoT (Industrial Internet of Things) began to thrive. Its business drive was promoted by the Industry 4.0 initiative, whereas its applications were extended from the very much overlapping Cyber-Physical Systems (CPS) domain. There is no generic, de-facto architecture for IIoT systems, although a layered approach is followed by domain experts. The purpose of splitting the layers could vary from communication types due to infrastructure need to the ecosystem stakeholder point of view; hence three, four or five layers can be identified. Figure 1 provides a layered architectural view which shows the strong separation of technologies between the layers. It also indicates the different security approaches at the different layers [1].

**Figure 2:**
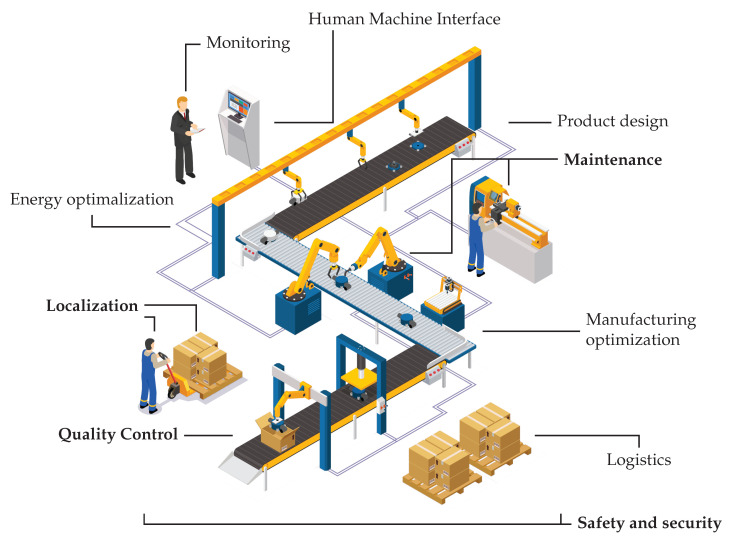
Production stages typically covered by the process of manufacturing. While machine learning can be applied in all areas of manufacturing, only a small subset of them use machine learning techniques extensively (shown in bold typeset).

While machine learning is exploited in various IIoT application areas, it is used only on a small subset of target areas extensively (see Figure 2). Depending on the application area, there are various purposes for processing industrial data. These include decision support, optimization, prediction, anomaly detection, classification, and clustering, just to name a few. In order to achieve the desired results, we need data—which are generally available for industrial players if IIoT-based data collection is in place—and we need physical resources for data processing—which are now available mostly due to the boom in GPU production. Because data and resources have been made available, we are able to use ML (Machine Learning) methods to achieve better results than ever before in the above areas.

In terms of finding details on these methods, the first resources to turn to are, naturally, textbooks. There are several great books on machine learning in general [2,3,4], and on modern tools regarding their application [5,6,7,8]. Further, we can find survey papers on utilizing machine learning in the industry. The authors of [9] provide a survey of the upcoming wave of machine learning in smart manufacturing. The specific topic of tackling faults by machine learning (ML) in the industry 4.0 era are surveyed in [10]. In a paper on machine learning multi-agent systems [11], the authors focus exclusively on their application in the oil and gas industry. Regarding different levels of industry 4.0, [12] focuses on ML methods applied in production planning and control. Similarly, a review of ML methods for the optimization of production processes is provided in [13].

To provide comparison with the topic of our current article, we can find more specific papers summarizing ML methods for smart production in general [14], or that review ML for production energy efficiency [15]. The authors of [16] provide a comprehensive overview of prognostic methods in the area of Industry 4.0. The authors of [17] focus on sustainability and predictive maintenance. Regarding reliability engineering and safety, the authors of [18] provide a targeted survey. Furthermore, ML support on safety assurance is surveyed in [19].

The main contribution of this paper is that it provides a structured state-of-the-art view of the domain, with well-structured comparison tables and details of the knowledge in this industry thus far. Although the area is very much an interest of industrial innovation, there is currently no structured, application-oriented overview available on machine learning methods in the Industrial Internet of Things (IIoT) domain. In particular, no complete overview of production quality, safety, maintenance and sustainability has been made available. Therefore, the aim of this current study is to fill this gap by providing a comprehensive overview of applied machine learning techniques within the aforementioned fields. Moreover, the paper also groups these applications by their main purposes to provide a deeper understanding of which techniques are used for certain typical tasks.

The structure of the paper is as follows. Each chapter addresses an application area, and provides a general overview of the issues and target solutions. Application examples are provided with the related ML methods. There are summary tables in each chapter, and a lessons learned section to highlight the main points. As such, Section 2 focuses on safety, security issues and solutions, Section 3 describes the main achievements in asset localization, Section 4 provides an overview of the methods and application use-cases for quality control, Section 5 targets maintenance and sustainability, and Section 6 concludes the paper.

## 2. Safety and Security

Industrial IoT is a convergence area of Operational Technology (OT) and Information Technology (IT), both of which contribute to the safety and security issues of Industrial IoT. Security and safety is undoubtedly one of the most important aspects of IIoT. To underline this, the Industry IoT Consortium has published a technical report [20] about the security issues in IIoT systems, summarizing all the experience and knowledge of the consortium.

**Figure 3:**
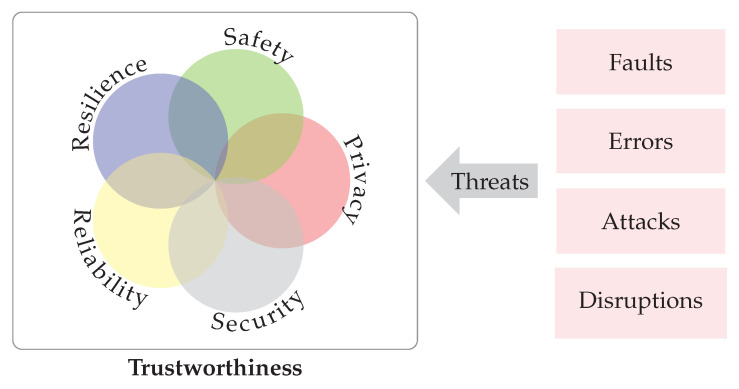
Trustworthiness of an IIoT System as specified by the Industrial IoT Consortium [20]. The key characteristics of the trustworthy IoT system are security, privacy, reliability, safety and resilience.

The main goal in IIoT systems is reaching the trustworthy status, where trustworthiness “is the degree of confidence one has that the system performs as expected in respect to all the key system characteristics in the face of environmental disruptions, human errors, system faults and attacks”. Figure 3 shows the key system characteristics of a trustworthy system when resisting external or internal threats. The key characteristics are [21]:

**Security**  —Security ensures that the system is protected from unintended or unauthorized access, change, or destruction.
**Privacy**  —Privacy provides organizations control over the collection, processing, and storage of their information, by deciding how this information can be shared both within their own organization and with others.
**Reliability**  —Reliability guarantees that the system’s operation is uninterrupted and error-free for the specified time. Availability is related to reliability, but also takes into account planned operation stops.
**Safety**  – System Safety ensures that the people, property and environment are not at any unacceptable risk during the system’s operation.
**Resilience**  —System resilience provides a way to dynamically avoid, absorb and rapidly recover from changing adverse conditions. Resilience includes the ability to withstand and recover from deliberate attacks, accidents, or naturally occurring threats or incidents.

While a secure and safe industrial IoT system requires appropriate system design, implementation and deployment, machine learning-based solutions are widely used to provide additional layers of security and safety. There are a couple of survey works that review the security issues of IIoT systems, mainly focusing on general security issues; however, machine learning-based solutions are presented as well in [1,22]. In [23], a layer-wise analysis of security issues and solutions are shown, specifically, in 5G-based IIoT systems. Moreover, ref. [24] applies a layer-wise approach; besides information on common security issues, it provides an in-depth overview of the security aspects of edge and fog computing.

While all the key characteristics of trustworthy IIoT systems are important, most of the machine learning-based solutions concern protection against unintended and unauthorized access (i.e., security). One of its typical application areas is *intrusion detection* in which the learning method tries to detect unauthorized access to the system from arbitrary features, e.g., system logs, monitoring services, etc. To achieve this, most works use supervised learning techniques (i.e., classification) to detect intrusion from features, e.g., k-nearest neighbour (kNN) [25], support vector machines (SVM) [26,27,28], decision trees [29], Bayes networks [30,31,32], random forests [33] and also neural networks [34,35,36]. Besides common classification solutions, fuzzy methods and association-based methods can be found [37]. In the past, hidden Markov models (HMM) were also proposed [38,39]. The subject of intrusion detection is so extensive that a couple of comprehensive survey papers focus on this topic alone [40,41]. While classification is widely applied to intrusion detection, intrusion can be seen as an outlier against authorized users. The survey provided in [42] reviews the application areas of outlier detection in IoT systems, summarizing examples of intrusion detection in the field of IIoT. Alongside intrusion detection, a wider level of *anomaly detection* can be acquired using machine learning in IIoT systems, by applying unsupervised or supervised methods to identify outliers or abnormal behavior in the system [43,44,45].

Strongly related to intrusion detection, *authentication* can be supported by machine learning techniques. Machine learning algorithms alone are rarely used for authentication purposes; however, it can provide an additional security layer over classical authentication schemes. Authentication based on network traffic analysis is performed in [46], using ensemble learning techniques. WiFi-capable IoT devices can be authenticated through the actuation of daily activities, as shown in [47]. Bregman divergence combined with the k-nearest neighbour technique makes it possible to detect Man-in-the-Middle attacks during authentication [48]. Furthermore, using stacked autoencoders (SAE) and k-means clustering, a high accuracy was reached in detecting WiFi impersonation attacks [49]. Blockchains play an important role in IIoT systems security; they are applied for various purposes, e.g., for distributed secure databases. The authentication of users to access blockchain is often integrated with a deep learning method that is taught by transfer learning [50,51]. Interestingly, authentication can be performed in the physical layer alone. Ref [25] proposes a solution to authenticate IoT devices with RF fingerprinting, using a software-defined radio (SDR) solution. The paper investigates different machine learning methods, kNN, SVM and decision trees, and all are proved to be accurate enough to perform authentication based solely on RF information.

*Privacy* is mostly ensured by encryption (using cryptography); however, there are certain issues regarding IIoT systems. While a couple of machine learning methods have been applied (e.g., in intrusion detection, authentication, etc.), the training of deep neural networks requires extensive datasets. Besides public datasets, it is commonly required to train the networks on distributed real datasets; however, this can result in the so called “privacy leaking”. There are solution and works that propose methods to avoid privacy leaking, e.g., privacy-preserving asynchronous deep learning schemes (DeepPAR [52]) or differential privacy and federated learning [53]. There are other methods for ensuring IIoT privacy using differential privacy; a thorough review can be found in [54].

*Data integrity* is a crucial part of a trustworthy IIoT system. Data integrity means that data are not modified over their lifetime; the data remain consistent and accurate. One of the most prevalent attacks against data integrity is false data injection (FDI); however, data integrity refers to all the possible combinations of data injection, data modification or even data relation disintegrity. The methods for data integrity checks commonly learn the distribution of valid data and identify outlier samples with a low likelihood. Data and the command injection were reviewed in [55], where a gas pipeline system remote terminal unit (RTU) was observed. Using six machine learning techniques (e.g., random forests, SVM, etc.), the injection attacks were accurately identified. Using k-means clustering, ref. [56] proposes a method for recognizing data modification in programmable logic controllers (PLC). In [57], deep belief networks and restricted Boltzmann-machines were applied to identify data injection attacks in smart grids. Using smart sensor data from a complex hydraulic IIoT system, autoencoders were trained to help avoid false data attacks in [58].

A reliable trustworthy IIoT systems requires high *availability*, meaning, that the system is ready to provide services to users. However, a common attack against IIoT devices is the so called denial-of-service (DoS) attack, which—by applying huge work load—prevents the device from providing services, and leads to it becoming temporarily unavailable. A common variation of this attack is the distributed DoS (DDoS) originating from a number of sources. Using a couple of network and log features, ref. [59] proposes a hybrid deep learning framework (deep belief networks, autoencoders, etc.) to classify the type of attack reaching the device, e.g., DoS attacks, among others. Using the game-theory approach, a reinforcement learning technique was proposed in [60] to identify DDoS attacks. Bayesian networks can also be successfully used to predict traffic delays and DDoS attacks; for example, in [61] the solution was inspired by the portfolio theory used in economics.

Industrial IoT systems have a special security issue, called *offload security*. Using machine learning algorithms in IIoT systems sometimes requires different calculations to be offloaded to edge devices, called edge or fog computing. This offloading gives rise to new kinds of security problems, since offloading tasks to the cloud or to the edge is vulnerable to security issues due to malicious devices. A typical solution uses blockchains and the reinforcement learning method to avoid the security problems of computation offloading, while implementing a double-dueling Q-network [62]. Other solutions typically use reinforcement learning methods, such as the solution in [63,64].

### 2.1. Datasets

There are a couple of public datasets available to train and validate machine learning algorithms in the topic of IIoT security. Reviewing these datasets can provide a deeper understanding of the machine learning algorithms, revealing the possible features and outcomes of each algorithm. The works presented before usually trained on these datasets.

One of the most famous intrusion detection datasets is the “The Third International Knowledge Discovery and Data Mining Tools Competition” KDD-99 dataset [65]. The dataset was prepared using the DARPA project, and contains 4 GB of network traffic from a period of seven weeks, containing attacks of four categories (DOS, R2L, U2R, probing).

The Canadian Institute for Cybersecurity provides a number of datasets regarding cyber security. The CSE-CIC-IDS2018 dataset [66] contains seven different attack scenarios in an infrastructure of 420 machines and 30 servers. Additionally, the institute provides a state-of-the-art dataset for detecting DDoS attacks [67].

Further datasets for intrusion detection and privacy attacks can be found in [68,69]. The University of Arizona provides datasets [70] for various security purposes, e.g., malware detection, intrusion detection, etc., as well.

### 2.2. Critics of Machine Learning Based Security

Zolanvari’s work [71] provided some criticisms regarding IIoT security and the datasets used for training purposes. Most machine learning-based algorithms and solutions for IIoT security (e.g., intrusion detection, DDoS prevention) need some data for the training and validation phases. For example, network traffic datasets require carefully selected features for the machine learning algorithms to function properly. If the features do not vary with the attack, the algorithm cannot be successful. Additionally, sensor data in IIoT applications are usually obtained with different sampling frequencies over an extended time, which results in high dimensional datasets. Using raw data such as this will lead to a large delay in training and detecting processes. Furthermore, it is hard to acquire real IIoT data from companies due to confidentiality and privacy restrictions; therefore, all the solutions presented typically are trained on the same publicly available datasets. Interestingly, the main problem with the available datasets is that the number of real attacks are significantly low compared to normal behavior; the highly unbalanced datasets make it hard to train learning algorithms effectively.

### 2.3. Summary of Security and Safety

Machine learning in IIoT security is widely used as an additional layer of security to provide a truly trustworthy system. However, the usually applied techniques are limited to a couple well-defined of use cases. The most important are the intrusion detection methods, or more generally, anomaly detection methods. Most works train and utilize SVM or Bayesian networks to recognize unusual behavior or—explicitly—intrusion. Numerous methods are also applied in the authentication process, even in the case of authentication without credentials, e.g., only in the physical layer. The other key area of machine learning algorithms in IIoT security is that of the detection and avoidance of DDoS attacks, using unsupervised techniques, e.g., autoencoders. A special area of IIoT security is the security issues resulting from offloading calculations in edge computing, i.e., the so called offload security. Machine learning methods require training to perform successfully, for which a couple of publicly available datasets are available; however, some researchers are against using these datasets to train and validate secure machine learning-based solutions. The references for different applications presented in this section are shown in Table 1.

## 3. Asset Localization

**Figure 4:**
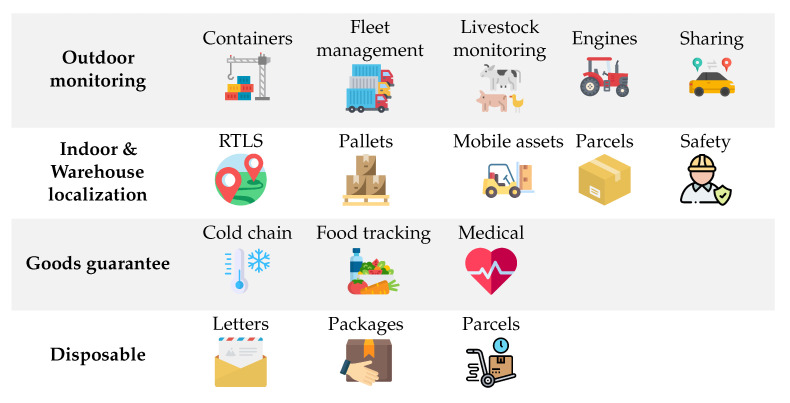
Typical use-cases for industrial indoor and outdoor asset tracking and localization. Beyond classical indoor and outdoor use-cases, there are a couple of less know topics, e.g., tracing the food chain or tracking disposable items (icons from Flaticon.com).

Asset localization is one of the most important and specialized features of IIoT systems, because either the manufacturing process or site security requires the location of—potentially semi-finished—products or assets to be tracked. There are a couple of typical use-cases for asset tracking and localization, which are depicted on Figure 4. While localization is mostly achieved with GPS (Global Positioning System) outdoor, it cannot be applied in indoor factories since the GPS signal is shadowed by the structure of the building. To overcome this issue, a couple of radio-based technologies are utilized and employed to provide a more or less accurate asset position in indoor situations. More specifically, all the radio technologies used in IoT or IIoT systems can provide measurements to acquire asset position; however, some solutions are more suitable for asset localization than others.

At first sight, localization is a geometric problem, because the properties of radio signal propagation can be calculated, i.e., the equations for the attenuation and the propagation delay are well-known. However, in indoor situations, especially on industrial sites, the dense multipath environment makes the propagation so random and stochastic that the received signal and its properties contain little information about the propagation. That is why a number of works apply complex machine learning methods to solve the localization problem instead of solving the geometrical problem. However, machine learning methods are also frequently used to improve the accuracy of the geometric solution.

### 3.1. UWB

Ultra-Wideband (UWB) technologies are widely used in IIoT solutions to provide accurate localization, since UWB—by design—is especially suited to localization purposes. The huge bandwidth (a couple of hundreds megahertz) of UWB makes it possible to apply very short pulses (e.g., 1–2 ns long) which helps to distinguish between the rays in multipath propagation to provide accurate timestamps for the received packets, resulting in a centimeter-capable localization. The method of location estimation in UWB systems is usually based on the calculation of the distances between anchors and tags (i.e., ranging); based on these calculated data, the geometric problem is solved by optimization. However, multipath propagation can distort the received signal, so errors in the timestamping process result in positioning errors.

To overcome this issue, ref. [72] investigates decision tree, random forest and kNN (k-nearest neighbour) algorithms to improve the accuracy of localization based purely on the calculated location information. A more sophisticated solution is to use the indicator provided by the receiver which helps to determine whether the timestamp belongs to the real first path (line-of-sight, LOS) or not (non line-of-sight, NLOS). Based on this information, ref. [73] uses a naive Bayes method to infer the improved position for the device.

In fact, machine learning methods are widely used in UWB systems to classify the reception as LOS or NLOS propagation. These methods are supported by the fact that most UWB chips provide the received channel impulse response (CIR) of the packet. Using CIR, the study in [74] trains a convolutional neural network to classify the reception of a packet as an LOS and NLOS reception, which helps the localization engine to weigh the measurement while calculating the position. Ref [75] also targets the classification of reception; however, it compares three machine learning techniques, namely support vector machines, random forests and dense neural networks. To make use of the temporal behaviour of the channel impulse response, ref. [76] investigates different combinations of convolutional neural networks and recurrent neural networks for CIR classification, showing that a CNN followed by stacked LSTM networks provides the best accuracy. The CIR can be used not only for classification, but for estimating the timestamping error of each of the received packets. Ref [77] uses convolutional networks to predict the error in timestamping based on the CIR of received packets, resulting in a one-order improvement in location accuracy on average compared to the geometric solution. The survey in [78] presents a couple of other UWB-based solutions, although not exclusively in the IIoT environment. A general approach for estimation of the position from CIR using deep learning techniques can be found in [79].

### 3.2. 5G

5G is a cutting-edge mobile technology, which supports on site installations besides large-scale infrastructure. In the 5G standard, great effort was made to improve the positioning capabilities of the previous LTE standard; since Release 17, IIoT localization has become an important topic of the standard [80]. 5G provides time- and angle-based positioning, and the variable parameters of the NR (New Radio) interface help to improve the accuracy of 5G positioning (e.g., higher bandwidth, variable subcarrier spacing, different antenna patterns, etc.).

While 5G supports different kinds of localization methods, these are mainly geometrical or closed-form solutions. However, a couple of works, which aim to improve the accuracy of the localization using machine learning methods, can be found—mainly concerning an IIoT environment, i.e., in indoor situations. One typical positioning method is “fingerprinting”, which is based on readily observable radio channel properties, such as the receive signal strength (RSSI). To improve the accuracy of such solutions, ref. [81] tries to estimate and correct the error of the location using different machine learning models. To estimate the position, the kNN technique and vanilla neural networks were compared. Targeting the same problem, ref. [82] compares kNN- and SVR- (Support Vector Regression) based techniques to the defined DELTA method, which implements a dense neural network to infer the position from RSSI measurements.

A more sophisticated method in 5G localization is angle-based positioning. Using beamforming, the technique in [83] samples the received PDPs (Power Delay Profile), creates beamformed fingerprints and uses trained temporal convolutional neural networks (TCN) and LSTM networks to infer the location from the beamformed fingerprint. The TCN network is capable of tracking the location with an accuracy of a couple of meters on average. The solution has low energy consumption, even compared to GPS systems. Also using deep neural networks, ref. [84] optimizes the handover process by improving 5G localization based on beamforming. The solution’s accuracy proved to be 1.25 m on average.

A comprehensive study on 5G and positioning can be found in [85], where a couple of methods—including machine learning-aided localization methods—are compared and introduced.

### 3.3. WiFi and Bluetooth Low Energy

The WiFi standard (IEEE 802.11) provides broadband communication technology, which is widely used in commercial and industrial areas. Without special hardware, WiFi localization solutions usually use fingerprinting techniques. To improve localization accuracy, a variety of trained machine learning models are used. The study in [86] compares the baseline kNN solution to the SVM and Random Forest techniques, while [87] integrated decision trees and naive Bayes methods to the comparison. The papers [88,89] use dense neural networks to learn fingerprint and localization mapping, while [90] utilizes denoising autoencoders to augment the received fingerprints and estimate the accurate location of the asset.

Bluetooth Low Energy (BLE) provides low-range, low-speed communication using low energy, and is widely used in a variety of areas. The basic localization technique in case of BLE is fingerprinting, and the same methods can be applied to improve the accuracy as in the WiFi case. However, there are works specifically related to BLE. Ref. [91] applies a special data augmentation process to train and evaluate random forest, XGBoost, decision tree and kNN-based algorithms for inferring the position from the RSSI measurements. The augmentation process helps learners to handle RSSI measurements with high fluctuation, thereby providing more accurate predictions. Ref. [92] is interesting as it introduces the applicability of the well-known LDA (Linear Discriminant Analysis) algorithm in fingerprinting. The work compares the LDA to naive Bayes, kNN and SVM techniques, showing an improvement in localization accuracy with a satisfactory execution time. A couple of other fingerprinting-based works can be found [93,94], and the study provided in [95] overviews the methods of fingerprinting in cases of BLE. Since the creation of Bluetooth 5.1, the BLE standard has made direction-of-arrival (DoA) methods available by using antenna arrays. Using the BLE DoA feature, [96] applies a tiny neural network applicable to a constrained device to replace the famous MUSIC (Multiple Signal Classification) algorithm when finding the signal direction.

### 3.4. Other

Apart from these widely used technologies, a couple of other solutions can be found in asset localization in IIoT systems. LOS/NLOS classification is shown in [97] for the less-common IEEE 802.15.4 systems, using SVM, random forests and neural networks. In [98], acoustic localization technology was employed in an IIoT underwater wireless sensor network, and linear regression was applied to predict the accuracy of node localization.

An increasing number of studies that analyze the applicability of the so-called device free localization (DFL), which provides user or asset location without any hardware attached, have been published. There are a couple of machine learning techniques utilized in those algorithms, e.g., block-sparse coding with the proximal operator [99] or Bayesian methods [100]. A useful summary of this topic and recent state-of-the-art can be found in [101,102].

### 3.5. Summary of Asset Localization

Asset localization in IIoT systems can be implemented using a variety of technologies, including UWB, 5G, WiFi, BLE, etc.; however, UWB is the only technology that specifically concentrates on localization. Machine learning methods are generally used for the following two purposes in case of each technology:

1. To learn the mapping between measurements and location
2. To improve the accuracy of the location deduced by closed-form, geometrical problems

The first is mainly used in case of fingerprinting—here, machine learning models try to learn the mapping between the measurements (mostly RSSI) and the location. The baseline solution is often the kNN learner; however, a couple of other regression methods are used, sometimes using classification on grids. The second one combines geometric models with machine learning models, where the following two basic methods can be distinguished: predicting LOS or NLOS propagation and predicting the localization error. The methods usually use additional information, e.g., the channel impulse response. The references for different applications presented in the section are shown in Table 2.

For further details, an in-depth study on indoor localization using machine learning techniques can be found in [103].

## 4. Quality Control

Quality control is a process by which entities review the quality of many factors involved in production. The primary responsibilities include monitoring, inspection, reducing product variation and eliminating failure cases. Functional and visual tests—often referred to as automated visual/surface inspection—are the two main steps of quality inspection in industrial manufacturing processes. Nowadays, both of these inspection categories are implemented by machines and not by humans in most cases; however, they rely on human expertise. Defective product detection demands well-defined quality requirements. To automate such quality inspection processes, these parameters have to be interpreted and tuned for specific automated identification processes, which is very challenging. Machine learning methods can help to overcome such difficulties.

### 4.1. Visual Quality Inspection

**Figure 5:**
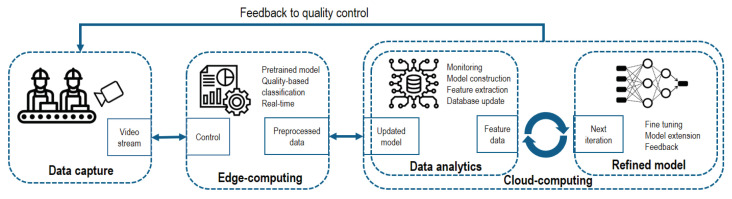
General architecture model for vision-based product quality inspection [104].

The existence of surface defects affects the product’s appearance and quality in many industry domains. Therefore, one of the most widely applied quality inspection methods in manufacturing is the visual inspection of some aspects of the product. There are many solutions for surface and external defect inspection in many domains of the industry, including the metal, semi-conductor and fiber industries. This section introduces the main directions of visual quality inspection methods supported by machine learning. Figure 5 presents the general model for vision-based product quality inspection according to [104].

In general, defective product identification requires two well-defined steps, i.e., feature extraction and defect identification. Product features could be in the spatial domain or in the transform domain. Moreover, there are several feature extraction methods used in the state-of-the-art, including classification (KNN, Naive Bayes classifier, SVM, Decision trees), clustering (K-means, PCA) and regression (Linear regression, logistic regression) methods. With the advent of deep-learning algorithms, a set of features no longer needs to be designed, such as statistical or spectral features, as opposed to traditional methods. There are several examples in the state-of-the-art surface inspection for supervised (CNN, LSTM) and unsupervised (Autoencoder) learning solutions.

Ref. [105] presents a computer-vision system using machine learning approach to inspect both the internal and external parts of aerospace components. SVM is used to classify defects of the external parts of the product, while for defect classification of the internal parts, a combination of CNN and LSTM is applied. In contrast, ref. [106] presents a quality-level estimation system for the inspection of steel microstructures using the VGG network model. Ref. [107] uses Convolutional Neural Networks (CNN) and Convolutional Autoencoders (CAE) for casting surface inspection. Ref. [108] provides a solution based on supervised learning to inspect press-casting products using CNN, Random Forest, PCA, and XGBoost. Similarly, ref. [109] examines the application of supervised machine learning (random forest, gradient boosting) in defect detection, quality assurance and throughput improvement for optical transceiver manufacturing. Ref. [110] proposes a method for error detection by applying a CNN model to the optical inspection of assembling machines. The CNN-based solution is used in [111] for classifying Pin-in-Paste solder connections with a YOLOv4 architecture. The framework contains highly automatized image data labeling functionality using a Convolutional Autoencoder and near real-time solder joint localization based on a YOLO single-stage detector. Ref. [112] introduces surface defect detection for metal workpieces. The paper introduces both the results of ResNet50 and DenseNet40 architectures. Ref. [104] uses CNN and SVM for defect classification and provides a machine vision model to identify defective products. Ref. [113] introduces semi-supervised deep learning-based surface inspection techniques of labeled data for automated surface defect inspection. Ref. [114] presents an unsupervised clustering method of spatial patterns with wafer map measurement data. Measured test values are first pre-processed using computer vision techniques, followed by feature extraction based on variational autoencoders to decompose high-dimensional wafer maps into a low-dimensional latent representation.

### 4.2. Anomaly Detection

The other significant quality inspection category is anomaly detection. The goal of anomaly detection—also referred to as outlier detection—is to determine all instances dissimilar to the others or the required instances. Ref. [115] defines an outlier as an observation that deviates so significantly from other observations as to arouse suspicion that a different mechanism generated it. Ref. [116] introduces an architecture where the machine learning algorithm can detect defective bearings and continually tunes the quality testing process parameters. Specifically, the identification of defective bearings is performed using a voting classifier fed by statistical metrics measured from the collected experiments. The paper evaluates several machine learning methods, including k-neighbors, SVC, Decision Tree, Random Forest, Multi-Layer Perceptron, AdaBoost, Naive Bayes, Gradient Boost, and Voting Classifier methods. Ref. [117] proposes a method in which (1) the manufacturing processes classification is performed using the Support Vector Machine (SVM) algorithm, (2) the regularization parameter value and the gamma coefficient value of the SVM algorithm are optimized using the Horse Optimization Algorithm (HOA), (3) the HOA -based SVM results are compared to Particle Swarm Optimization (PSO)-based SVM results and Chicken Swarm Optimization (CSO)-based SVM results. Additionally, ref. [118] uses PSO and DNN for a similar problem. Both methods are validated on SEMCOM dataset [119].

Without application dependability or a case study, ref. [120] introduces an anomaly detection method based on the Gaussian Restricted Boltzmann Machine for industry product quality inspection. Ref. [121] addresses the critical issues of machine learning-based condition monitoring solutions. Ref. [122] studies several unsupervised learning techniques (Gaussian model, SVM, isolation forest, autoencoder) based on six industrial test datasets. Ref. [123] focuses on a particular application of telemetry—anomaly detection on time-series data. It presents an improved version of ReRe, a state-of-the-art Long Short Term Memory-based machine learning algorithm.

A fuzzy neural network-based fault diagnosis approach for condition monitoring is presented by [124] for rotating machines via vibration signals. Ref. [125] proposes a long short-term memory (LSTM)-Gauss-NBayes method for outlier detection in the IIoT. LSTM-NN builds a model on a normal time series and it detects outliers by utilizing the predictive error for the Gaussian Naive Bayes model. Ref. [126] proposes an on-device federated learning-based deep anomaly detection framework for sensing time-series data in IIoT. The framework used an attention mechanism-based convolutional neural network-long short-term memory (AMCNN-LSTM) model to detect anomalies accurately. Similarly, ref. [127] proposes a reliable anomaly detection strategy for IIoT using federated learning. Specifically, it applies the federated learning technique to build a universal anomaly detection model with each local model trained by the deep reinforcement learning algorithm.

In contrast, ref. [128] introduces a practical investigation on graph neural networks (GNNs) for anomaly detection in IIoT-enabled smart transportation, smart energy, and smart factory. Ref. [127] designs an anomaly detection algorithm that exploits deep learning techniques to assess the working conditions of the plant. AE and deepAE are responsible for initial dimensionality reduction, PCA is responsible for a further reduction, and K-means clustering performs the actual anomaly detection. Ref. [129] develops a methodology for detecting abnormal behavior in the context of aging IIoT using a PCA-based method.

### 4.3. Datasets for Anomaly Detection

There are a couple of public datasets that can be used to train and validate machine learning algorithms on IIoT quality inspection and outlier detection. Reviewing these datasets can provide a deeper understanding of the machine learning algorithms, revealing the possible features and outcomes of each algorithm. Ref. [119] provides a collection of databases, domain theories, and data generators that are used by the machine learning community for the empirical analysis of machine learning algorithms. There are several datasets from the manufacturing domain that are used for algorithm validation, including the semi-conductor domain. Ref. [130] provides access to a large collection of outlier detection datasets with ground truth (if available). The focus of the repository is to provide datasets from different domains and present them under a single platform for the research community, including several manufacturing domains (wafer map).

### 4.4. Summary of Machine Learning Based Quality Control

Visual quality inspection and surface detection are the most common quality inspection methods and are utilized in almost every domain of the industry and in manufacturing (see Table 3). Pretrained CNN networks (ResNet, DenseNet, VGG) can be leveraged for object recognition, while Autoencoders are used for feature extraction.

Since device failures seriously affect the production of industrial products in Industrial IoT (IIoT), accurate and even real-time anomaly detection is becoming increasingly important. Due to the nature of IIoT, federated learning solutions are used in many industrial domains. LSTM networks gained much attention as they are well-suited to classifying, processing and making predictions based on time series data, which are one of the most common data sources in anomaly detection problems.

## 5. Maintenance

Maintenance has always been an important aspect of industrial manufacturing as it includes crucial tasks that directly affect productivity. Traditionally, maintenance involves the following two key factors: the cost of repairing or replacing equipment and the cost of shutting down production lines when the required equipment or tools are unavailable. Therefore, maintenance evolves alongside industrial technologies and new approaches.

Two different approaches are reactiveand proactive maintenance. Reactive maintenance refers to the intuitive way of maintenance, which means that the maintenance task will be performed after an items wears-out or is broken. Proactive maintenance involves different methodologies to actively monitor the equipment, create strategies, and estimate the conditions in order to prevent failure to save cost by ensuring shorter-term and scheduled maintenance and longer operational capabilities.

**Figure 6:**
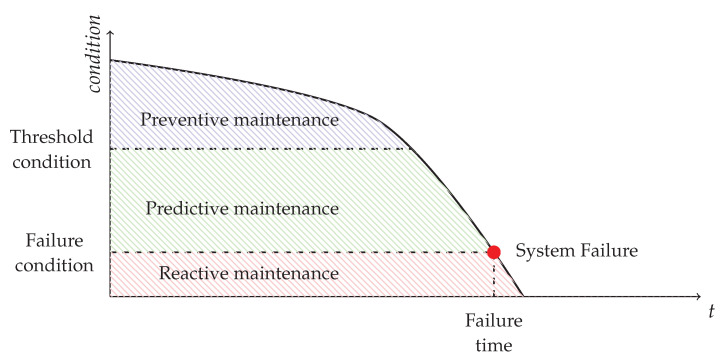
Difference between maintenance approaches in terms of condition and time.

The most widely adopted methodologies are predictive and preventive maintenance. While the first places emphasis on estimating the time of failure to enable scheduled maintenance—and therefore production line down-times—the other aims to establish a regular, periodic maintenance process for preventing failures and keeping machinery operational for as long as possible by expanding its life-time [131,132,133], as shown in Figure 6.

Usually, proactive maintenance refers to the application of predictive with preventive approaches in the same maintenance ecosystem to overcome the disadvantages of wasting working hours and costs by performing unnecessary, periodic maintenance.

### 5.1. Tasks of Proactive Maintenance

Industry 4.0 applications that usually involve IoT systems as well are not only the enablers of proactive maintenance; they also benefit from adopting it. This leads us to the concept of Cyber–physical systems that include numerous interconnected subsystems on a physical and a digital plane. Such a CPS collects data about the status and conditions of the subsystems or pieces of equipment which can be used for optimizing certain parameters of the system, thereby creating a feedback loop. This scheme can be also applied for maintenance tasks in order to create an CPS/IoT-enabled proactive maintenance system. Such a system consists of several main areas that can be classified as follows [134,135,136]:

**Fault detection**  — Detecting malfunctions is a complex task which involves several data sources such as equipment monitoring sensors, environment monitoring sensors, telemetry data, etc., in order to be able to recognize failures. The most common data that are gathered by sensors are: vibration monitoring, sound or acoustic monitoring and oil-analysis or lubricant monitoring [137,138].
**Diagnostics**  — Diagnostic processes are at the core of prognostics and strategy planning as they provide an analysis of failures and hazards, thus enabling the creation of models. One of the main task of diagnostics is Root cause analysis, which is a framework for investigating hazards and systematically discovering the possible root causes [139,140,141].
**Prognostics**  — The aim of prognostics is to estimate the future condition of equipment by modelling it based on the results of diagnostics. In most cases, the final goal of prognostics is to calculate the Remaining Useful Life (RUL) and Mean Time to Failure (MTTF). These factors play a key role in predicting and preventing possible future malfunctions and failures and help to schedule required maintenance tasks in time [142].

As discussed, these areas of maintenance include tasks such as data analysis, pattern recognition, designing complex models of processes or object, and forecasting events (hazards and failures), which are areas where machine learning techniques traditionally outperform other methods and solutions. As the fields of maintenance, repair, and overhaul (MRO) do not have any strict regulations, state-of-the-art solutions can be constructed based on requirements and expectations, and they can also follow the already implemented solutions available in the literature or as an open-source project.

### 5.2. Fault Detection

Each area of maintenance requires a huge amount of data; Fault detection (FD) and anomaly detection are not exceptions. This task shares many solutions with the aforementioned anomaly detection method in quality control, due to their common nature. Since the main goal is to observe and identify failures, classification, clustering, regression and anomaly detection algorithms are best suited for this use-case [143].

In [144], a clustering-based solution is discussed for the fault detection of a Power Distribution Network, where the decision tree algorithm outperformed KNN and SVM in terms of accuracy. In [145], the authors proposed a clustering approach for detecting multi-component degradation in aircraft fuel systems. Decision tree-based solutions, such as the one detailed in [146,147], are also capable of providing an accurate detection rate, while maintaining a low computational complexity.

Nonetheless, using neural networks for this purpose is also a common solution, such as in [148], where a completed maintenance framework is built upon it. Artificial Neural Network-based solutions can be effectively used for feature extraction and for analysis time-series data, as is shown in [149,150]. Moreover, fault detection is the one of the main tasks of predictive maintenance that strongly relies on real-time computation. Ref. [151] presents a Convolutional Neural Network (CNN)-based solution for motor fault detection that can provide accurate estimations in real time.

### 5.3. Diagnostics

Since diagnostic tasks, most prominently Root cause analysis, investigate hazards by systematically creating problem subsets, classification or clustering based solutions are often required.

In [152], the authors proposed a Decision Tree and Principal Component Analysis (PCA)-based RCA solution for rotating machinery, where PCA can eliminate the redundant features, while Decision Tree can classify data with high precision. Ref. [153] also showed that Decision Tree and Ensemble algorithms perform the best for large amounts of data with uncertain problems. For the specific rotating machinery use-case, [154] applied Random Forest and KNN as classifiers within their proposed methodological framework for time-series data, while [155] also used Random Forest for time-domain classification.

RCA is not the only important diagnostic task, as diagnostics also includes modelling that serves as the basis for prognostics. Such tasks usually requires feature extraction to build models. In [156], the authors proposed a solution for rotating machinery fault diagnosis based on auto-encoder to perform feature extraction and a fish swarm algorithm to optimize its key parameters. Ref. [157] also proposes a similar method for rolling bearings, but with an enhanced Deep Wavelet Auto-encoder and Extreme Learning Machine. For bearings, different CNN-based solutions were introduced in [158,159] to process time and frequency series data from sensors. Another solution is presented in [157] using RNN and GRU for high accuracy and robust performance.

### 5.4. Prognostics

Besides estimating RUL and MTTF, prognostics usually involves design or create models that can describe the behaviour of the investigated equipment or component. There are numerous approaches that can be applied in this field, namely Physical model-based, knowledge-based, data-driven, etc., models, among which data-driven methods are now predominant due to the huge amount of available data and emerging machine learning applications. Nonetheless, certain learning techniques such as fuzzy logic can also be applied for knowledge-based predictions [160].

Regarding the data-driven approach in [161], a Support Vector Machine (SVM) and Restricted Boltzmann Machine (RBM)-based solution is proposed for estimating RUL. In this work, a dataset measured using a vibration sensor was classified with SVM, while RBM was used to enable learning without abnormal data. Such a functional combination is typical within this field, where one technique is applied for classification while the other is used for to overcome unbalanced data. According to [162], SVM is one of the most popular algorithms for classification; however, but statistical algorithms such as Bayesian Networks are still relevant, mostly in uncertain environments or in the case of a small amount of data [163,164].

A typical problem in modelling complex systems is the massive state space that makes it difficult to create a model that takes each feature into account. Therefore, reducing complexity is a common task in this field, where auto-encoder-based [165] solutions can be intensively applied. In [166], the authors proposed an Auto-Encoder Gated Recurrent Unit (GRU) for dimension reduction before calculating RUL; in [167], it was applied the same way, but within a framework.

Since such predictions are mostly based on time-series data, it is usually beneficial to implement systems that can deal with this type of data. Ref. [168] applied Long Short-Term Memory (LSTM) and Recurrent Neural Network (RNN) for High-speed railway power equipment, due to its powerful prediction ability for time-series. In [169], a combined LTSM-RNN solution was applied in the same manner; however, the study pointed out that it is suitable for only critical systems due its complexity, while for a huge amount of data vanilla-RNN is more suitable.

### 5.5. Manufacturing Optimization

This section introduces the key aspects and machine learning methods used for manufacturing optimization. It covers only the processes that are directly part of the production line and the manufacturing processes. The target variables of the manufacturing optimization process include the quality of the product, cost, time, power consumption or other product-specific parameters. Naturally, there are correlations between these optimization factors, yet, these are the most notable categories. The use of machine learning techniques is highly beneficial to pattern recognition, which is the core of the manufacturing optimization processes. With the help of ML techniques, correlations between different types of data or manufacturing domains can be identified and utilized to optimize the manufacturing process.

For electricity optimization, ref. [170] uses Q-learning in an automation system to reduce electricity consumption. In ref. [171], a mixed online bipartite matching-based Deep Q-network algorithm is proposed for profit-maximizing smart manufacturing. The paper formulates a joint optimization of the block size, task scheduling, and supply–demand configuration to maximize customers’ net profit with the probabilistic delay requirements, which addresses the critical issue of efficiency and latency in the blockchain-based live manufacturing process. Conversely, the study in [172] uses a support vector regression algorithm with an RBF kernel for troubleshooting production data to identify parameters responsible for high energy conversion efficiency variances. Ref. [173] propose LithoGAN, an end-to-end lithography modeling framework based on a generative adversarial network (GAN), to map the input mask patterns directly to the output resist patterns. The results show that LithoGAN can predict resist patterns with high accuracy while achieving a speed that is orders of magnitude greater than conventional lithography simulation and previous machine learning-based approaches. Besides GAN and Q-learning, there are CNN and RNN applications in the field of ultrasound imaging. Ref. [174] proposes an automatic fetal ultrasound standard plane recognition model in an IIoT environment which learns the spatial and temporal features of the ultrasound video stream by using multi-task learning. The CNN component identifies fetal key anatomical structures, while the RNN component obtains the temporal information between adjacent frames, and it realizes the precise localization and tracking of fetal organs across frames.

Particle Swarm Optimization has been utilized in several optimization problems; ref. [175] proposes a PSO-based technique to optimize the hyperparameter settings of the LSTM in an FL environment, while in [176] combined multi-Objective particle swarm optimization (CMOPSO) is proposed for a green manufacturing energy system. Regression problems are also quite typical in the field of manufacturing optimization. Ref. [177] proposes optimizing semiconductor manufacturing processes through machine learning (ML) based on a regression algorithm. Ref. [178] compares the performance of different supervised machine learning methods for the field calibration of low-cost IoT sensors, including Linear Regression and Artificial Neural Network solutions. Furthermore, ref. [179] presents a method of using logistic regression to solve the inverse problem in electrical impedance tomography. However, there are generally fully connected NN solutions in the manufacturing industry. Ref. [180] proposes Finite Element Analysis and a NN model to optimize and create chip package design. Ref. [181] introduces an optimization method for Bipolar-CMOS-DMOS process development based on an Automatic Multi-objective Optimization solution and NN.

There are a couple of survey works in the field of manufacturing optimization. The study in [13] covers the majority of relevant literature from 2008 to 2018 concerned with machine learning and optimization approaches for product quality or process improvement in the the manufacturing industry. The review shows that there is hardly any correlation between the used data, the amount of data, the machine learning algorithms, the used optimizers, and the respective problem from the production. Additionally, there are works summarizing some of the recent advancements in ML with a focus on its applications in the process industries [182,183], such as in additive manufacturing [184]. Furthermore, ref. [185] summarize the designs of state and action, provides RL-based algorithms for scheduling, and reviews the applications of RL for different types of scheduling problems.

### 5.6. Datasets for Smart Maintenance

There are a couple of public datasets that can help to train and validate machine learning algorithms in smart maintenance tasks. Reviewing these datasets can provide a deeper understanding of the machine learning algorithms, revealing the possible features and outcomes of each algorithm. Given the lack of real, industrial data, most of these datasets are synthetic. As fault detection is involved both in quality control and maintenance, ref. [119] is also a useful source for smart maintenance applications. Ref. [186] uses MetroPT, a benchmark dataset for predictive maintenance collected in 2022 about an urban metro public transportation service in Porto. The data contain samples from analog sensor signals (pressure, temperature, current consumption), digital signals (control signals, discrete signals), and GPS information (latitude, longitude, and speed) for anomaly detection and failure-prediction purposes. Ref. [187] provides a dataset that consists of a sequence of alarms logged by packaging equipment in an industrial environment for classification, forecasting and anomaly detection purposes. The collection includes data logged by 20 machines, and deployed in different plants around the world, from 21 February 2019 to 17 June 2020. There are 154 distinct alarm codes, for which the distribution is highly unbalanced.

### 5.7. The MANTIS Proactive Maintenance Platform

**Figure 7:**
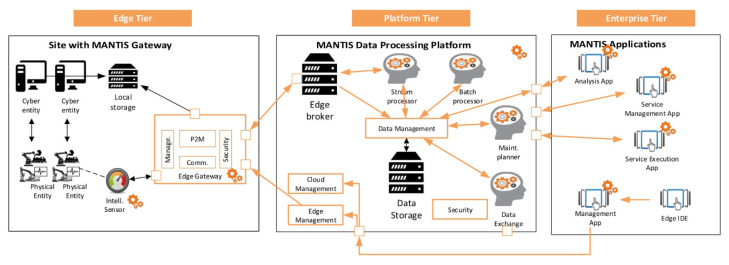
Overview of the MANTIS reference architecture [188].

The MANTIS project of Electronic Components and Systems for European Leadership started in 2015 with 47 different partners across Europe from 12 different countries. In [188], the authors detail that the main objective was to develop a CPS-based Proactive Maintenance Service Platform Architecture enabling Collaborative Maintenance Ecosystems. The requirements were set to match the expectations for optimising maintenance mechanisms for CPS [189]. They proposed the following four main proactive maintenance target areas: the Remaining Useful Life (RUL) of components, Fault Prediction (FP), Root Cause Analysis (RCA), and Maintenance Strategy Optimization (MSO). The architecture model follows the Industrial Internet of Things Reference Architecture of Industrial Internet Consortium and contains the following three main tiers: the edge tier, platform tier, and enterprise tier, as can be seen in Figure 7, and it also supports multi-stakeholder interactions, while the architecture has been validated by different evaluations. MANTIS uses the Lambda architecture pattern for data processing and it uses the Open Standards for the Physical Asset Management of Machinery Information Management Open System Alliance (MIMOSA) for common understanding, databus, and data ontology between partners and applications.

In [190], the authors present an extension of MANTIS including a Big Data implementation and technologies, such as the Hadoop Distributed File System, Apache Spark. They used two techniques, namely Root Cause Analysis powered by Attribute Oriented Induction (AOI) Clustering and Remaining Useful Life based on time series forecasting. In the platform technological implementation, the following four main blocks are explained: Data Access and Ingestion through the Edge Broker, Data Storage systems, Batch Processor, and Human Machine Interfaces.

In [191], the authors detailed CPS-populated systems used for proactive maintenance using MANTIS. They introduced the main characteristics of a CPS and summarized three main research challenges, namely science and engineering foundations; system performance, quality and acceptance; and applied development and deployment. They also presented the interoperability perspective of MANTIS, including the specification, conceptual integration, application integration, and technical integration aspects.

In [192,193], the authors present complex case studies on continuous monitoring and proactive maintenance of the railroad tracks and railway switches. For the whole process, they used the concepts and the platform of MANTIS. They detailed the data-processing steps, implementation approaches and visualization solutions highlighting the advantages of the usage of MANTIS in each step.

### 5.8. Summary of Machine Learning Based Maintenance and Manufacturing Optimization

The main areas of proactive maintenance—fault detection, diagnostics and prognostics —share some common requirements and targets and, consequently, the tasks that need to be performed. These key tasks are usually classification, clustering, regression, complexity reduction, system modelling, and data series analysis. However, there are specific targets for these areas as the former ones are more closely related to fault detection and diagnostics while the latter ones are related to prognostics; the applied machine-learning techniques also overlap to a certain degree. A summary of the methods used in maintenance can be found in Table 4.

For clustering, KNN and SVM are typically used, while for classification and regression purposes decision trees, random forest and principal component analysis are used. The most popular techniques for modelling, complexity reduction, and data series analysis are neural network-based applications including CNN, auto-encoder, RNN, GRU and LSTM.

Naturally, there are very broad, application-specific optimization problems in the manufacturing industry and differences in their regularities in the context of the used machine learning techniques. Supervised machine learning techniques (regression, SVM) are used for prediction and as an analytical tool during the optimization process in most cases for classification purposes. Reinforcement learning, especially Q-learning, is utilized in several instances during manufacturing processes for decision-making problems such as single and multi-objective scheduling problems.

However, it can be concluded that there is only a fine margin between manufacturing optimization and quality inspection. In many cases, the two processes cannot be separated; there are several dependencies between such machine learning-supported manufacturing processes.

## 6. Conclusions

This paper provided a comprehensive overview on machine learning techniques applied for various purposes in IIoT and Smart Production. The domains covered include safety and security, asset localization, quality control, and proactive maintenance.

IIoT security and safety is a very important aspect for the Industry 4.0 technology transition; hence, one can find many different application domains for ML techniques. These include the identification of intrusion detection, supporting authentication, realizing privacy leaking, checking data integrity, supporting availability, and offloading security services. Asset localization is a very specific area of smart production, where machine learning has been applied extensively. The application areas for asset localization include learning mapping between measurements and location, predicting non-LOS propagation, and predicting location error. Regarding quality control, visual quality inspection and anomaly detection applications were found to specifically require machine learning approaches. In relation to maintenance, the main application areas include fault detection, diagnostics, prognostics, and some manufacturing optimization applications which were surveyed too.

Besides providing a general overview of the applied techniques for the listed application areas, this paper also summarized the related references found for application domains, making it easier for practitioners and researchers alike to find ML-application patterns for their given field. The paper also contains the most important references of public datasets for developing domain specific algorithms and applications (see Table 5). Each main chapter includes a dedicated lessons-learned section to reinforce the main findings about the state-of-the-art and the research gaps of the discussed application area.

## Figures and Tables

**Table 1:** Summary of applications of machine learning techniques in IIoT security and safety.

Application	Typical Machine Learning Techniques	References
Intrusion detection	Classification on network data (SVM, Bayes networks, decision tree, Random forest, neural network)	[25,26,27,28,29,30,31,32,33,34,35,36,37,38,39,40,41,42]
Authentication	Classification on network data, Clustering	[25,46,47,48,49,50,51]
Privacy leaking	Differential privacy and federated learning	[52,53,54]
Data integrity	Latent space methods (Boltzmann-machine, DBN), Classification (Random Forest, SVM)	[55,56,57,58]
Availability	Reinforcement learning and Neural networks (DBN, autoencoders)	[59,60,61]
Offload security	Reinforcement learning	[62,63,64]

**Table 2:** Summary of applications of machine learning techniques in IIoT asset localization.

Application	Typical Machine Learning Techniques	References
Learning mapping between measurements and location	kNN, SVM, Random Forest, XGBoost, Regression tree, neural networks, etc.	[79,82,83,86,87,88,89,90,91,92,93,94,95,96]
Predicting non-LOS propagation	Neural network (CNN, TCN, etc.), SVM, Random Forests on channel impulse response	[72,73,74,75,76]
Predicting location error	Neural network on channel impulse	[77,81,98]

**Table 3:** Summary of applications of machine learning techniques in IIoT quality control.

Application	Typical Machine Learning Techniques	References
Visual quality inspection	CNN (Yolo, VGG, ResNet, DenseNet), Autoencoders	[104,106,107,110,111,112,114]
Anomaly detection	LSTM and PSO, kNN, SVM, PCA, XGBoost, Regressions, etc.	[117,122,125,126,127,129,129]

**Table 4:** Summary of applications of machine learning techniques in IIoT proactive maintenance.

Application	Typical Machine Learning Techniques	References
Fault Detection	KNN, SVM, Decision Tree, CNN	[143,144,145,146,147,148,149,150,151]
Diagnostics	Decision Tree, Random Forest, KNN, SVM, CNN, RNN	[152,153,154,155,156,157,158,159,157]
Prognostics	SVM, Bayesian Networks, RNN, CNN, Auto-Encoder, LSTM, Gated Recurrent Unit (GRM)	[160,161,162,163,164,165,166,167,168,169]
Manufacturing optimization	Unsupervised learning (Regressions, SVM, GAN), Reinforcement learning (Q-learning, LSTM)	[170,171,173,174,175,177,178,181]

**Table 5:** Summary of major and typical datasets for IIoT machine learning applications.

Topic	Name of Dataset	Description
Smart maintenance	MetroPT [186]	Consists of samples of analog sensor signals (pressure, temperature, current consumption), digital signals (control signals, discrete signals), and GPS information (latitude, longitude, and speed).
	Alarm Logs in Packaging Industry (ALPI) [187]	Contains a sequence of alarms logged by packaging equipment in an industrial environment. The collection includes data logged by 20 machines, deployed in different plants around the world, from 21 February 2019 to 17 June 2020.
Quality inspection	UCI Machine Learning Repository [119]	A UCI collection of databases, domain theories, and data generators. There are several datasets from the manufacturing domain that are used for algorithm validation, including the semi-conductor domain.
	Outlier Detection DataSets [130]	ODDS provide access to a large collection of outlier detection datasets with ground truth (if available). The focus of the repository is to provide datasets from different domains including several manufacturing domains (wafer map).
Safety and security	KDD-99 dataset [65]	The dataset used for The Third International Knowledge Discovery and Data Mining Tools Competition, the competition task was to build a network intrusion detector algorithm.
	CSE-CIC-IDS2018 dataset [66]	The dataset includes seven different attack scenarios, namely Brute-force, Heartbleed, Botnet, DoS, DDoS, Web attacks, and infiltration of the network from inside. The attacking infrastructure includes 50 machines and the victim organization has 5 departments including 420 PCs and 30 servers.
	CIC DDoS attack dataset [67]	The dataset contains different modern reflective DDoS attacks such as PortMap, NetBIOS, LDAP, MSSQL, UDP, UDP-Lag, SYN, NTP, DNS and SNMP.
	Intrusion detection and privacy attack dataset [68,69]	Dataset for developing and evaluating different IEEE 802.11 Wi-Fi algorithms.
	The University of Arizona datasets [70]	Different malware and network traffic datasets for developing and evaluating network security algorithms.
Localization	UTIL: An Ultra-wideband Time-difference-of-arrival Indoor Localization Dataset [194]	An Ultra-wideband Time-difference-of-arrival Indoor Localization Dataset. Raw sensor data including UWB TDOA, inertial measurement unit (IMU), optical flow, time-of-flight (ToF) laser, and millimeter-accurate ground truth data were collected during the flights of drones.
	CSI Dataset towards 5G NR High-Precision Positioning [195]	This dataset can be used for indoor positioning, indoor-outdoor-integrated positioning, NLoS, 5G channel estimation and other types of research, providing researchers with CSI-level position-related feature data.
